# Advancing early access policies for innovative cancer drugs: a scoping review and explorative analysis in the Italian setting

**DOI:** 10.1080/20523211.2024.2377697

**Published:** 2024-07-15

**Authors:** Margherita d’Errico, Diana Giannarelli, Daniela d’Angela, Carmine Pinto, Barbara Polistena, Federico Spandonaro

**Affiliations:** a C.R.E.A. Sanità, University of Rome Tor Vergata, Rome, Italy; b Medical Oncology, Comprehensive Cancer Centre, AUSL-IRCCS di Reggio Emilia, Reggio Emilia, Italy

**Keywords:** Early access; neoplasms; economic evaluation; health policy; innovative medicines; review

## Abstract

**Introduction:**

Considering the clinical impact of innovative cancer therapies, policy makers strive to balance timely access and thorough value-assessment. While some European countries promoted early access schemes, Italy does not yet display a consolidated strategy for innovative drugs or for medicines targeting pathologies with a high unmet need.

**Methods:**

To better understand the risks and opportunities of early access strategies that could be applied in the Italian setting, we performed a scoping review, searching the PubMed and Web of Science databases and interviewing two field experts. The review results were complemented with an exemplificative quantitative analysis for a subset of innovative oncology drugs, to assess the clinical and economic impact of the price and reimbursement negotiation.

**Results:**

Our study suggests that early access schemes developed in Germany and France, combining a free-pricing period, pay-back mechanism, and arbitration, could serve as a basis for developing a feasible strategy in Italy. The quantitative analysis indicated that timely access to innovative drugs could have potentially prevented many cancer progressions, associated with a significant healthcare expenditure.

**Conclusion:**

Albeit not allowing to express a conclusive assessment, this study proposes a potential early access strategy for Italy and highlights the need for opening a debate on the opportunities and risks of such schemes.

## Introduction

The scientific advancements of the last decades and the development of innovative therapies brought to the reality of clinical practice new therapeutic opportunities, often determining a turning point in the treatment of life-threatening diseases, among which numerous cancers (Schilsky et al., 2020).

The peculiarity of innovative therapies, in terms of clinical outcomes and organisational impact, requires specific arrangements between manufacturers and payers at the time of market authorisation (Masini et al., 2021). In this regard, the market access for pharmaceutical products is strongly regulated worldwide: although with some differences, most countries developed a Price & Reimbursement (P&R) strategy with overlapping characteristics, based on consolidated methodologies of clinical and economic evaluation (Martinalbo et al., 2016). The assessment process and, in some jurisdictions, the price negotiation, after the European Medical Agency (EMA) centralised approval, requires a technical time that slows the market access of new medicines.

**Figure 1.**
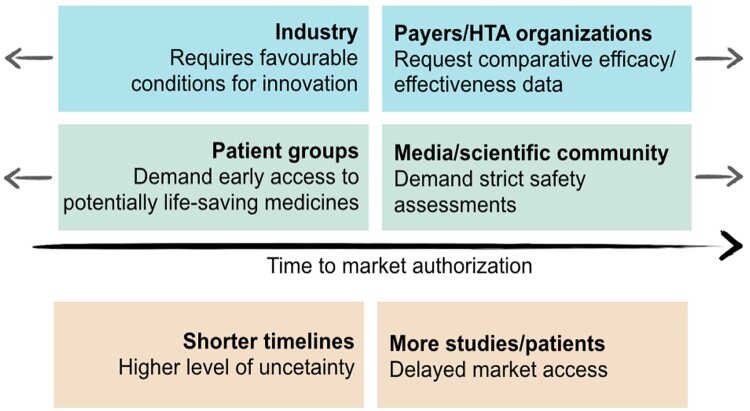
Different driving forces in the Price & Reimbursement (P&R) process, leading to market authorisation, adapted from Eichler et al. (2008)

In Italy, the P&R process efficiency improved over the years and progressively reduced its duration (Montilla et al., 2022). However, the challenges posed by clinical outcomes uncertainty and sustainability place an implicit limit on further reductions of the process duration. Considering the undoubted benefits of new drugs, a trade-off between timeliness and accuracy of the necessary assessments is deemed fundamental. On one hand, patients are interested in accessing life-saving therapies as soon as possible, while the pharmaceutical industry encourages a rapid access to foster innovation; on the other hand, regulatory authorities must conduct their assessments with the necessary accuracy (Figure 1) (Eichler et al., 2008; Panteli et al., 2016).

To address this trade-off, many European countries promoted early access (EA) schemes, mainly aiming to reduce the time to market access (Pang et al., 2019). In this regard, a recent report listed Italy as the 2nd among European countries for the number of medicines made available to patients, while it drops to 15th place when considering the time between market authorisation and access to the reimbursement list, with a meantime of 436 days (Newton et al., 2023). Italy thus presents a 308-day delay compared to Germany (128 days), where no negotiation is foreseen at the time of market access (Martinalbo et al., 2016; Newton et al., 2023). Based on recent information provided by the Italian Medicines Agency (AIFA) and the analysis of the drug approval publications in the Italian Official Journal, the time between EMA approval and the Italian P&R ‘*Determina*' ranges between 4.0 and 26.8 months (Montilla et al., 2022; C.R.E.A. Sanità Data on File).

With this data suggesting the relevancy of EAs scheme, recent times have seen a growing debate on its risks and opportunities. For example, the paper by Tarantola et al. compares EA programmes adopted in France, Spain, and the United Kingdom, analysing its implications for the Italian setting (Tarantola et al., 2023). They however seem to focus on early access schemes where medicines are covered by 3rd payers for accelerating access of drugs targeting an important unmet need. In this context, our study focuses on early access programmes that can be applied after market-authorisation, addressing EAs schemes that can minimise the cost attributable to the technical time of the P&R process, particularly evaluating from a health policy perspective the implications of introducing EA programmes for innovative drugs or for medicines targeting pathologies with a high unmet need. For this purpose, a scoping review was conducted to describe the current policy framework in Europe and to evaluate potential strategies that could be adopted in Italy, considering its peculiarities and different regulations. Additionally, an illustrative quantitative analysis was carried out on a subset of innovative cancer drugs, to better understand the clinical and economic implications of delayed access to therapy in Italy.

## Methods

### Research design

A mixed-method study was conducted to describe EA programmes in Europe, as well as estimate the burden associated with delayed access to therapy in Italy. First, a scoping review was performed to identify the state of the art of early access solutions adopted internationally, taking as an example France and Germany, that were pioneers in the field (BfArM, 2024; Degrassat-Théas et al., 2015). After assessing the political challenges resulting from the introduction of EA strategies for innovative cancer drugs, a model was developed to attempt a quantification of the clinical and economic impact of the delayed access to care, considering innovative cancer drugs that have been already approved by the Italian Medical agency (AIFA).

### Scoping review

The scoping review focused on EA strategies granting access to medicines with an EMA authorisation without, or before, the completion of a P&R process; the review considered strategies developed in Europe, later focusing on Germany and France, because these two countries presented a regulatory framework coherent with the goal of accelerate access, without undermining financial sustainability. This scoping review approach was chosen as it is suitable for identifying current best practices in a relatively new or developing field, and it was developed following the six-step framework by Arksey and O'Malley (2005). After identifying and selecting relevant sources with a broad search strategy, data was reported with a narrative approach. Additional references were provided through a consultation with field-experts as described below.

#### Search strategy, eligibility criteria, and data reporting

The PubMed and Web of Science databases were searched from January 2015 to March 2022, to include only the most updated publications on the topic. The search strategy included text words and Medical Subject Headings (MeSH) related to ‘early access strategies' and ‘innovative therapies' and it is reported in Supplemental Material File S1.

Title and abstract of the identified articles were screened and selected based on the following inclusion criteria: (1) publications on EA strategies applicable to therapies already authorised by EMA, that did not yet receive a P&R decision, (2) publications on future perspectives aimed to shorten the medicines time-to-availability, and exclusion criteria: (3) publication not reporting EA strategies relevant to France or Germany; (4) publications investigating access to therapy prior to EMA registration, (5) publications on the role of managed-entry agreements (MEAs) in the standard P&R negotiation, (6) publications analysing single approvals or a specific drug group, seven publications for which an updated version was available. The search was extended to editorials, institutional web publications and reports, laws, and official gazettes, published in English, French, and German. Data was extracted based on the following fields: author, publication year, country, article type, main findings. Key findings were summarised thematically.

#### Semi-structured interviews

The review findings were validated and integrated by conducting two semi-structured interviews with two health economists that were expert in the field. Interviews enabled the inclusion of the most updated insights on country-specific regulatory frameworks and future perspectives and facilitated access to grey literature and records published in languages other than English. One field expert from France and one from Germany, both chosen via convenience sampling, were prompted to answer 21 open-ended questions covering the following topics: (1) generalities of the authorisation to patient access in each country and stakeholders involved, (2) local care delivery and medicinal products supply, (3) P&R process, (4) negotiation process for innovative and orphan drugs, (5) EA strategies and MEAs. The topic list was developed after the scoping review of key findings. The interviews were conducted in English by one researcher (MD) and performed following a protocol adapted from the Standards for Reporting Qualitative Research (SRQR) guideline (O'Brien et al., 2014). Oral informed consent for the interview recording was obtained at the meeting beginning, following a description of the interview structure and duration (1.5 h). The two semi-structured interview were conducted on March 31, and April 11, 2022 respectively, using the digital platform Teams.

#### Interview processing and data analysis

In parallel to audio recordings, supportive notes were taken during the interviews to ensure the completeness of the collected information. Interviews were transcribed verbatim, adopting an edited transcription method, that implies the omission of words or sentences considered not relevant or repetitive, while maintaining the essential meaning and structure of the text (Guest & MacQueen, 2008). Considering the inclusion of only two participants, no encoding was performed on interview transcriptions, that were organised according to five topic areas: (1) overview and involved stakeholders, (2) healthcare delivery in the territory, (3) P&R negotiation process, (4) specificities of innovative and orphan drugs, (5) early access strategies and MEAs.

To ensure the credibility and trustworthiness of the analysis, two researchers assessed the interview transcription independently (MD, DG), addressing possible discrepancies of preliminary results, later sent to the interviewees for accuracy checking (Doyle, 2007).

### Epidemiological model

The analysis considered a subset of medicines already approved by AIFA, labelled as ‘innovative' as of October 2022 (AIFA, 2022). Of note, the approval for reimbursement by AIFA and the innovation classification implies that the medicine in questions successfully underwent analyses of cost-effectiveness and budget impact. Moreover, the new drug must have been associated with a therapeutical added value in a primary endpoint.

For the purpose of this exemplificative analysis, we focused on the first-line treatment of solid tumours and selected 4 drugs with 4 respective indications, from a total of 8 drugs (10 indications). Our choice aimed to include two targeted therapies and two immunotherapies, and to represent patient populations with different impacts. Additionally, if a drug was indicated for multiple types of cancer, only one indication per drug was included.

The target population for each drug was determined by retrieving data on cancer prevalence in Italy using national reports or published literature. Progression-free survival (PFS) data were retrieved from the relevant clinical trials and the number of preventable cancer progressions was calculated by multiplying the target population by the percentage of yearly PFS gain adjusted by the duration of the P&R negotiation process in Italy for each included drug. PFS was chosen for the calculations because, while not being affected by cross-over nor by different therapeutic sequencing, constituted the primary (or co-primary) endpoint for most of the considered pivotal studies. Overall survival data was not available for several trials at the time of the analysis. Estimates of drug efficacy were taken from the relative pivotal studies and when necessary derived from the published plots using the Digitize software.

### Economic assessment

The economic burden associated with delayed access to therapy was estimated multiplying the number of patients experiencing a disease advancement in the time window required for the P&R negotiation by the pro-capita costs that have been associated with cancer progression. These costs were calculated using estimates specific to Italy, identified by performing a snowball literature search. Calculations were repeated for each treatment pathway. The analysis considered the months between the granting of an AIC code by EMA to the publication in the Italian Official Journal and accounted for direct healthcare costs, adopting the perspective of the Italian national healthcare system (INHS). All costs were adjusted to 2023 using the CCEMG-EEPI-Centre cost converter, a web-based tool recommended by the WHO.

## Results

### Scoping review

The search identified 1325 articles plus 14 records identified through other sources (grey literature searching, snowball searching, national reports, citation searching, expert consultation) (Supplemental Figure S1). After title and abstract screening and exclusion of records not fulfilling the eligibility criteria, 29 articles were retrieved for full-text assessment and 10 were included in the final review (Supplemental Table S2). Table 2 summarises the EA strategies that directly or indirectly facilitate early access to therapy in the included countries.

#### Germany

**Figure 2.**
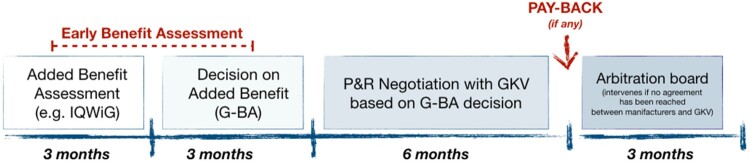
The HTA process in Germany from EMA approval to negotiated price.

The German Health Technology Assessment (HTA) procedure follows the Pharmaceuticals Market Reorganisation Act (Arzneimittelmarkt-Neuordnungsgesetz, AMNOG) (Figure 2). After EMA registration, manufacturers are invited to send a dossier to the Federal Joint Committee (*Gemeinsamer Bundesausschuss*, G-BA), responsible for the ‘early benefit assessment' with the support of the Institute for Quality and Efficiency in Health Care (IQWiG) during the first six months after submission (IQWiG, 2022). This first phase ends with the G-BA granting the status of ‘added benefit' or ‘no added benefit'. Regardless of the G-BA decision, in the following six months the manufacturers and the head association of the statutory health insurance (*Gesetzliche Krankenversicherung*, GKV) face four price negotiation rounds. The first 12 months are characterised by free-pricing for manufacturers and full reimbursement by the GKV (GKV-SV, 2022a, 2022c). Not reaching an agreement results in three more months of negotiation mediated by a *super partes* arbitration board. If the negotiated price is lower than the initial one, manufacturers are not obligated to pay back the difference between the initial launch price and the price determined after AMNOG negotiations. However, in case of intervention of the arbitrary board, the price is dated back to the 12th commercialisation month. Concerns about the overall strategy sustainability may result in shortening the free-pricing phase to 6 months, as proposed by the present government.

#### France

The HTA process of medicines newly approved by EMA in France is mainly mediated by the French National Health Authority (*Haute Autoritè de Santè*, HAS), in collaboration with the Transparency Commission (TC), that provides a technical assessment of the new drug, and the economic and public health commission (*Commission d’ évaluation économique et de santé publique*, CEESP) (CEPS, 2021). The recommendation produced by the TC and the CEESP is evaluated by the French medicine pricing committee (*Comité Economique des Produits de Santé*, CEPS), representing the main authority of the P&R negotiations (CEPS, 2021).

Regarding early access to therapies, France established the Temporary Authorisation for Use (ATU) programme (Albin et al., 2019; Martinalbo et al., 2016; Schleich et al., 2019) whose former six extensions have been grouped into two tracks since July 2021: the ‘Early Access’ (*Access Prècoce*) and the ‘Compassionate use' (*Access Compassionnel*) programmes (CEPS, 2021; Chu et al., Réforme de l’accès dérogatoire aux médicaments./2022; HAS, 2022). Among the extensions of the former ATU programme, the post-ATU (post-AMM) is a transitory designation that facilitated continuity of care for patients receiving an ATU therapy, covering the months between EMA approval and P&R decision (HAS, 2021). This function is currently included in the *Access Prècoce* programme and financed by the French Social Security’s sickness fund, at a price set by the manufacturer. New medicines may access this fast track by fulfilling criteria assessing product innovation, unmet need, disease rarity and severity. The EA designation is granted for one year, plus a possible one-year extension, and the therapy delivery usually initiates the calculation of the pay-back, firstly based on the sales recorded, and later on the difference between the initial price set by the manufacturer and the negotiated price (HAS, 2021, 2022). Notably, this strategy enables the collection of real-world data useful to address the clinical uncertainties of new therapies. Regarding the shortening of time-to-availability for all other medicines (i.e. non innovative), the CEPS committed to keep the negotiation timeline to a maximum of 120 days. Among possible future developments, there is a proposal from the LEEM (*Les enterprises du médicament*) addressed also in the 2022 social security system financial law. The LEEM proposed for drugs that obtained a recommendation by the TC a system comparable to the German AMNOG, namely direct access to patients, free-pricing and full reimbursement. The P&R negotiations would start in parallel and be limited to 12 months. More specifically, after 9 months the CEPS would come up with a price proposal, which, if rejected by the manufacturer, would be followed by three months of arbitrary process.

### Epidemiological model

The following paragraphs provide results specific for the pathologies included in the study and their targeting drugs, selected from the most recent official list of innovative medicines made available by AIFA at the time of the analysis. The detailed calculations are reported in Supplemental Material Table S1.

#### Breast cancer (BC)

In the analysis was included abemaciclib, indicated for the first-line treatment of positive Hormone-Receptor (HR+) and Her2 negative (Her-) breast cancer. The calculation of potentially avoidable cases started from the 834,200 prevalent cases reported by the Italian Cancer Association (AIOM) in 2022 (AIOM, 2022). The 5.6% of 834,200 prevalent cases was assumed as the prevalence of metastatic breast cancer (mBC), (Crocetti et al., 2018). Next, the molecular subtype distribution was derived from the North American Association of Central Cancer Registries (NAACCR), namely 73.0% of cases with both HR+ and Her-, and 15.0% of patients with Her2+ breast cancer irrespective of HR status (NAACCR, 2019). These hypotheses resulted in estimating 34,102 mBC patients eligible for treatment with abemaciclib per year. A yearly PFS gain of +11.9% was calculated from the pivotal study that supported the first-line use of abemaciclib (Monarch 3 trial), estimating 7102 avoidable progressions considering the P&R duration recorded in Italy, namely 14.7 months (Goetz et al., 2017).

#### Hepatocellular carcinoma (HCC)

The only innovative treatment in Italy for HCC at the time of the analysis was the combination of atezolizumab and bevacizumab. The number of avoidable progressions was estimated starting from the 12,100 yearly new diagnoses reported by AIOM (AIOM, 2022). Around 8.3% of these cases has an initial diagnosis of stage IV cancer, resulting in 1000 eligible patients (AIOM, 2022). Based on the PFS reported in the relevant clinical trial, a total of 461 avoidable progressions was estimated in the 20.5 months of negotiation (Finn et al., 2020).

#### MSI colorectal cancer (MSI CRC)

Treatment with pembrolizumab was the only MSI CRC innovative therapy listed by AIFA. Considering the 48,100 incident cases reported by the AIOM in 2022 and a reported incidence of stage IV cases equal to 1.1%, a total of 550 cases were included in the analysis. Considering a PFS of 18.0% (KEYNOTE-177 trial), a total of 404 avoidable progressions in the 14.0 months of P&R negotiation in Italy was estimated (André et al., 2020).

#### Non-small cell lung cancer (NSCLC)

The considered drug osimertinib was designated as innovative for the first-line treatment of EGFR positive (EGFR+) NSCLC. In 2021, AIOM reported 11,400 metastatic not squamous incident NSCLC. As they represent 70.0% of total mNSCLC cases (expert opinion), this was estimated to consist of 16,286 total cases. The remaining 30.0% thus represents the number of metastatic squamous NSCLC cases, namely 4886. According to the 2018 AIOM guidelines, the incidence of EGFR+ cases is 15.0%, resulting in 1710 EGFR+ cases (Facchinetti et al., 2019). The absolute gain in 1-year PFS with osimertinib is 25.4% (FLAURA trial), resulting in 651 avoidable progressions considering the negotiation months (Soria et al., 2018).

### Economic assessment

This economic evaluation accounted for direct healthcare costs associated with cancer progression in patients affected by BC, HCC, MSI CRC, and NSCLC.

#### Breast cancer

Pro-capita healthcare costs associated with BC progression amounted to € 6993.0 and were estimated using the costs reported by Mennini et al., subtracting the costs of patients in progression from the primary breast cancer patients not in progression (Mennini et al., 2021). Considering costs attributable to the technical time of negotiation of abemaciclib, i.e. costs due to potentially avoidable progressions, we estimated a total expenditure of € 49.7 mln (Table 1).

**Table 1.** Total costs attributable to progression of cancer progressions occurring during the negotiation process in Italy.

Drug name	Cost increase (€)	Avoidable cases (N)	Total costs (€, mln.)
Breast cancer*			
Abemaciclib	6993.0*	7102^p^	49.7
*Hepatocellular carcinoma*°			
Atezolizumab + bevacizumab	3285.7	461^p^	1.5
*MSI colorectal cancer*˟			
Pembrolizumab	9052.6	404^p^	3.7
*Non-small cell lung cancer*ˠ			
Osimertinib	6207.7°	651^p^	4.0

mln. = million; p = progression; r = recurrence.

*Costs derived by Mennini et al. (2021); ˠcosts derived by Buja et al. (2021); ˟costs derived by Francisci et al. (2013); °costs derived by Colombo et al. (2015).

#### Hepatocellular carcinoma

Costs associated to HCC progressions were calculated starting from the figures reported by Colombo et al. for patients with advanced HCC to which we applied the ratios between early and advance cancer costs extracted from White et al. (Colombo et al., 2015; White et al., 2012). Overall, a cost of € 3285.7 was assumed to constitute pro-capita costs associated with HCC progression. The total costs associated with the P&R process of atezolizumab and bevacizumab, and thus potentially preventable by granting early access to these drugs, amounted to € 1.5 mln (Table 1).

#### MSI colorectal cancer

Pro-capita healthcare costs associated with MSI CRC progression amounted to € 9052.6 and were calculated subtracting the costs of patients with stage I/II and stage IV MSI CRC derived from Francisci et al. (2013). Considering costs attributable to the technical time of negotiation, i.e. costs due to potentially avoidable progressions, € 3.7 mln were attributed to the negotiation technical time of pembrolizumab (Table 1).

#### Non-small cell lung cancer

Stage I and Stage IV NSCLC patient pro-capita costs in Italy were retrieved from Buja et al. and employed to estimate progression-attributable costs (Buja et al., 2021). A pro-capita cost of € 6207.71 was multiplied by the number of potentially avoidable progressions that were estimated to occur in the time window between EMA approval and publication in the Italian Official Journal. Negotiation of the treatment with osimertinib was associated with total preventable costs equal to € 4.0 mln (Table 1).

## Discussion

In Italy, the debate on the challenges and opportunities of EA schemes has just recently started. Currently, the literature does not provide enough information on operational insight that could contribute to the development and implementation of a strategy, aimed to minimise the cost attributable to the technical time of the P&R process, applicable in the Italian setting, that represents our study aim.

For example, a recent study comprehensively described EA programmes in Italy, France, Spain, and the UK (Tarantola et al., 2023) but did not consider Germany which completes the P&R procedures in the first year after market access, representing a relevant case for the above-mentioned purpose.

For this reason, a scoping review was conducted to identify and describe EA schemes adopted in other EU countries and potentially applicable to Italy. In particular, the analysis included Germany and France and assessed the transferability of their framework in the Italian setting, while excluding the UK, whose EA scheme seems rather focused on therapy access before EMA authorisation.

The review allowed to gather information on schemes adopted by Germany and France, which were seemingly implemented without significantly impacting the overall economic sustainability of the healthcare system (Table 2). More specifically, in Germany all drugs initially enter the market at a price set by the manufacturer; a benefit assessment, based substantially on elements derivable from the clinical practice, postponing the price negotiation (IQWiG, 2022).

**Table 2.** Early Access strategies that facilitate access to therapies with an EMA registration, before the end of P&R negotiations in France and Germany.

Country	Level	Responsible agency	Early access strategy	Eligibility criteria	Payer	Pay-back
France	Programme, Individual patient/cohort	HAS (TC, CEPS, CEESP)	Accès Précoce (i.e. all post-MA ATU) = 1 year (+ 1 yr extension), free pricing, full reimbursement during clinical data collection	ATU status, innovative drugs, for rare, severe diseases, high unmet need	CNAM	Yes sales-based discounts, negotiated price difference
Germany	Whole framework	IQWiG, G-BA	AMNOG procedure = 1-year free pricing, full reimbursement (6 months of benefit assessment + 6 months price negotiation)	All products with an EMA registration	GKV	No*

AIFA = Agenzia Italiana del Farmaco; AMNOG = Arzneimittelmarkt-Neuordnungsgesetz; ATU = Authorisations Temporaires d’Utilisation; CEESP = Commission d’ évaluation économique et de santé publique; CEPS = Comité Economique des Produits de Santé; CNAM = Caisse nationale de l'Assurance Maladie; G-BA = Gemeinsamer Bundesausschuss; GKV = Gesetzliche Krankenversicherung; HAS = Haute Autorité de Santé; IQWiG = Institut für Qualität und Wirtschaftlichkeit im Gesundheitswesen; MA = Market Authorisation; MHRA = Medicines and Healthcare products Regulatory Agency; TC = Transparency Commission.

*A mechanism of payback applies if the negotiation exceeds 12 months.

Of note, potential future developments in Germany include the possible reduction of the free-pricing phase, with the introduction of a pay-back mechanism after the 6th month of early benefit assessment.

France presents an EA scheme that is somewhat superimposable to the German one, albeit limited to innovative drugs for severe and rare diseases (GKV-SV, 2022b, 2022c).

Additionally, the French scheme addresses the issue of financial sustainability by foreseeing a pay-back mechanism, i.e. the return of the turnover share attributable to the difference between the manufacturer launch price and the one subsequently negotiated.

EA schemes currently implemented are in line with patients’ expectations – namely the ‘immediate' access to innovative therapies – and largely with those of manufacturers. While regulatory authorities must consider the risk of these schemes on the system’s overall sustainability, this risk appears to be largely mitigated by pay-back mechanisms and the provision of a limited negotiation time.

Regulatory authorities could also fear a loss of bargaining power during negotiation, as revoking a reimbursement decision for a drug already widely used is deemed less likely to happen, also in the case of a failed negotiation agreement. A point of interest, that requires further research, is the introduction of a *super partes* arbitration mechanism like in the German AMNOG procedure. An arbitration, excluding situations of *empasse*, could represents a valid response to potential losses in bargaining power.

To better understand the burden associated with a delayed access to therapy in Italy, a quantitative analysis was conducted on a subset of cancer drugs approved in Italy and labelled by AIFA as innovative.

Total costs related to the technical time of negotiation were estimated multiplying pro-capita costs by the number of progressions estimated to occur during the P&R negotiations. Exemplificative elaborations were developed for a set of innovative drugs and resulted in estimating a potential saving of € 49.7 mln for BC patients in progression, € 1.5 mln for HCC, € 3.7 for MSI CRC, and € 4.0 mln for NSCLC (Table 1). Notably, the figures here presented refer to potential savings for the INHS, as the actual impact of early access to these medicines highly depends on the outcome of the negotiation process. The calculation of avoided costs due to early access was, in fact, based on the costs of disease progression. However, this does not necessarily translate into equivalent saving for the INHS. The INHS would pay for the drug in advance, hypothetically at a price set by the industry, as in Germany, and that may differ from the negotiated price later on, impacting the overall savings.

It is also important to mention that the study did not consider costs per QALY gained but rather focused on the cases of progressions and relapses that could have been avoided by granting patients an earlier access to these medicines. The main reason for this choice is that all the included medicines have been already approved by AIFA and have been listed among the reimbursed drugs in Italy. This indicates that the cost-effectiveness and cost-utility, as well as the sustainability in terms of budget impact of these drugs has been already assessed, thus already determining the value for money of these medical products.

While using PFS measures, due to the current absence of OS data, does not account for the outcome of patients that will eventually progress, it is important to notice that a timely treatment might result in a higher number of patients becoming disease free or having a better prognosis. Also, since a drug is approved and defined as innovative by AIFA based on efficacy measures for primary endpoints, the PFS of the included drugs has been likely expected to be, at least partially, translated in an OS gain. Although these results refer to a subset of the innovative drugs approved in Italy, they seem sufficient to appreciate the potential impact of an EA scheme. The benefits of an EA programme extended to all new drugs, or at least to all the new medicines with an ‘innovation' status, would likely exceed the overall benefit here presented. While this study contributed with a number of insights to the discussion on Early Access, it is important to address a number of limitations that characterise this research design. First, it is assumed that the general target population of an Early Access programme of the included drugs would behave as the study population of the clinical trials, posing the issue of generalisability. This choice was however necessary due to the lack of real-world data for the innovative medicines considered in the analysis.

Also, the present study considered the time window between EMA approval and publication in the Italian Official Journal for the calculations. However, even the introduction of an EA scheme could not fully eliminate certain administrative procedures (e.g. obtaining an AIC number, shipment, and distribution of the medicinal product). Therefore, the adoption of this timeframe might have resulted in a cost overestimation. Another limitation of the model is the inclusion of exclusively oncology drugs considered innovative by AIFA and targeting solid tumours that shall be addressed in future research by including a comprehensive set of medicines.

## Conclusion

Although these results do not allow to express a conclusive assessment, they underline the importance of shortening the time to availability for innovative drugs, at least in Italy, despite the Italian P&R process appearing relatively rapid compared to other European countries.

Observing the EA strategies implemented by France and Germany demonstrated the feasibility of schemes that favour rapid market access of new medicines and that are compatible with the necessary guarantee of financial sustainability, mainly thanks to the adoption of short-term payback mechanisms. A possible concern of public authorities is the risk of losing bargaining power during negotiation, that seems however addressable with an arbitration process.

This study also attempted, for the first time to our knowledge, to quantify the human and economic costs of the technical time of negotiation in Italy and provided evidence on best practices adopted in Europe to overcome the trade-off between timeliness and accuracy.

## Supplementary Material

Supplementary_material_revised cleaned.docx
