# Supplementary material for: Advancing early access policies for innovative cancer drugs: a scoping review and explorative analysis in the Italian setting
Source: J Pharm Policy Pract. 2024 Jul 15;17(1):2377697. doi: 10.1080/20523211.2024.2377697 (PMC11251434; doi:10.1080/20523211.2024.2377697)
Supplement: Supplementary_material_revised cleaned.docx [file JPPP_A_2377697_SM5394.docx]

**Supplementary material**

| Supplementary Table 1: Number of breast cancer progressions or recurrence occurring during negotiation process in Italy | | | | | | | | |
| --- | --- | --- | --- | --- | --- | --- | --- | --- |
| **Cancer type** | **Drug** | **Eligibility** | **Prevalent mBC (N)** | **HR+/Her-** | **HR+Her-  per year (N)** | **Yearly  PFS gain** | **Negotiation time  ITA (months)** | **Avoidable progressions ITA** |
| BC | abemaciclib | HR+/Her- | 46,715 | 73.0% | 34,102 | 17.0% | 14.7 | 7,102 |
| **Cancer type** | **Drug** | **Eligibility** | **Incident cases (N)** | **advanced naive (%)** | **advanced naive (N)** | **Yearly  PFS gain** | **Negotiation time  ITA (months)** | **Avoidable progressions ITA** |
| HCC | atezolizumab + bevacizumab | advanced  naive | 12,000 | 8.3% | 1,000 | 27.0% | 20.5 | 461 |
| **Cancer type** | **Drug** | **Eligibility** | **Incident cases (N)** | **stage IV**  **(%)** | **stage IV**  **(N)** | **Yearly  PFS gain** | **Negotiation time  ITA (months)** | **Avoidable progressions ITA** |
| MSI CRC | pembrolizumab | Stage IV | 48,100 | 1.1% | 550 | 18.0% | 14.0 | 404 |
| **Cancer type** | **Drug** | **Eligibility** | **Incident mNSCLC (N)** | **EGFR+ (%)** | **EGFR+ (N)** | **Yearly  PFS gain** | **Negotiation time  ITA (months)** | **Avoidable progressions ITA** |
| NSCLC | osimertinib | EGFR+ | 11,400 | 15.0% | 1,710 | 25.4% | 18.0 | 651 |
| *BC = breast cancer; mBC = metastatic breast cancer; HCC = hepatocellular carcinoma; MSI CRC = microsatellite instability colorectal cancer; NSCLC = non-small cell lung cancer; mNSCLC = metastatic non-small cell lung cancer* | | | | | | | | |

| Supplementary Figure 1: Scoping review flowchart adapted from the PRISMA Statement (2020 updated version) |
| --- |
| 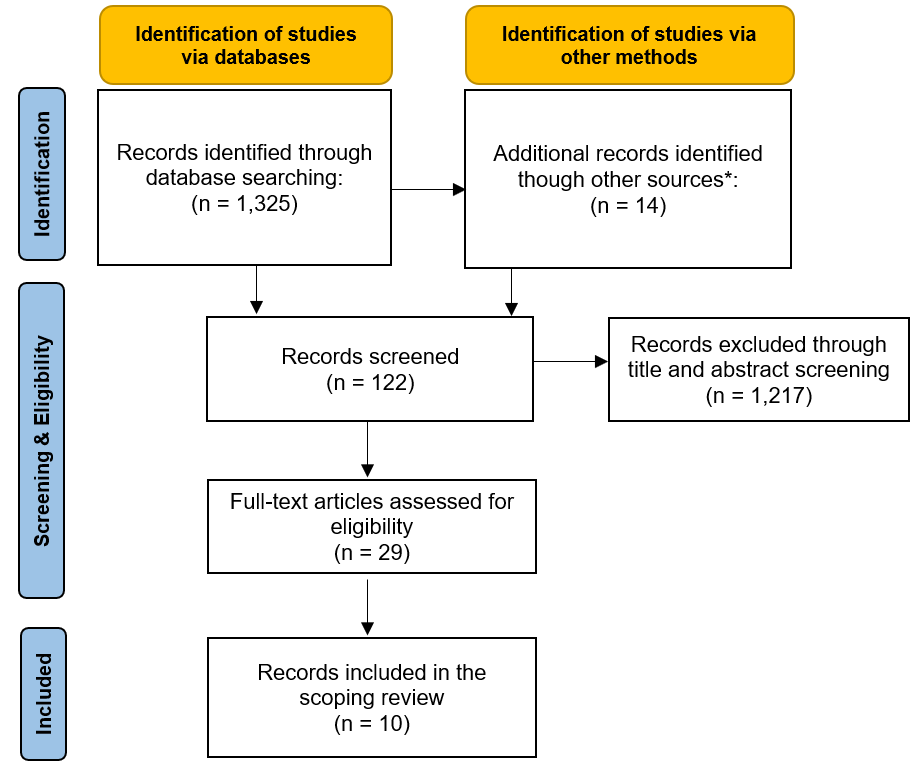 |
| **other sources = grey literature searching, snowball searching, national reports, citation searching, expert consultation*  © C.R.E.A. Sanità |

| Supplementary Table 2: Articles that were included or excluded after the full-text reading and data extraction performed in the scoping review. The reason for exclusion as well as the relevant eligibility criterium are indicated for each excluded paper. | | | |
| --- | --- | --- | --- |
| **Included articles** | | | |
| Refer to the bibliography: Albin et al., 2019; CEPS 2021; Chu et al., 2022; GKV-SV 2022a,b; GKV-SV 2022c; HAS 2021; HAS 2022; IQWiG 2022; Martinalbo et al., 2016; Schleich et al., 2019 | | | |
| **Excluded articles** |  |  |  |
| Author | Year | Reason for exclusion | Eligibility criterium* |
| Aapro et al. | 2017 | Not reporting EA strategies relevant to France or Germany | 3) |
| Balasubramanian et al. | 2016 | Focused on compassionate use programs | 4) |
| Bouvy et al. | 2018 | Focused on managed entry agreements | 5) |
| Buyukkaramikli et al. | 2021 | Focused on managed entry agreements | 5) |
| Capozzi et al. | 2018 | Not reporting EA strategies relevant to France or Germany | 3) |
| Fens et al. | 2021 | Focused on managed entry agreements | 5) |
| Ferrario et al. | 2017 | Not reporting EA strategies relevant to France or Germany | 3) |
| Gamba et al. | 2020 | Focused on managed entry agreements | 5) |
| Godman et al. | 2018 | Not reporting EA strategies relevant to France or Germany | 3) |
| Godman et al. | 2021 | Focused on managed entry agreements | 5) |
| Jørgensen et al. | 2017 | Focused on managed entry agreements | 5) |
| Leyens et al. | 2016 | Not reporting EA strategies relevant to France or Germany | 3) |
| Newton et al. | 2021 | A more updated version of the report is available | 7) |
| Patikorn et al. | 2021 | Focused on managed entry agreements | 5) |
| Pham et al. | 2022 | Analysing a specific pathological group (solid tumours) | 6) |
| Russo et al. | 2021 | Focused on managed entry agreements | 5) |
| Villa et al. | 2018 | Not reporting EA strategies relevant to France or Germany | 3) |
| Wettstein et al. | 2019 | Not reporting EA strategies relevant to France or Germany | 3) |
| Xoxi et al. | 2021 | Not reporting EA strategies relevant to France or Germany | 3) |
| **Referring to the eligibility criteria described in the methods section*  *EA = early access* | | |  |

| Supplementary File 1: Search strategy adopted for the scoping review |
| --- |
| ("early market access" [tw] OR "early access" [tw] OR "early patient access" [tw] OR "early access program*" [tw] OR "Managed entry agreement*" [tw] OR "accelerated approval" [tw]) AND ("Therapies, Investigational"[Mesh] OR “innovative therap*” [tw] OR "Drug Approval"[Mesh] OR “cancer drug*” [tw] OR "Antineoplastic Agents"[Mesh] OR “medicine*” [tw] OR “therapy” [tw] OR “therapeutic*” [tw]). Additional relevant hits were identified searching national databases and performing cross-reference searching. The articles were imported in EndNote X9 and duplicates removed manually. |
